# Medication‐Related Osteonecrosis of the Jaw After Dental Extractions During High‐Dose Denosumab Therapy in a Patient With Paget's Disease: A Case Report

**DOI:** 10.1002/ccr3.73659

**Published:** 2026-11-03

**Authors:** Toktam Zamani, Pegah Mosannen Mozaffari, Kazem Anvari, Najmeh Anbiaee, Khashayar Famili, Ali Mirzaei, Fatemeh Fallah Toosi, Abolfazl Shafiee

**Affiliations:** ^1^ Oral & Maxillofacial Diseases Research Center Mashhad University of Medical Sciences Mashhad Iran; ^2^ Oral and Maxillofacial Medicine Scientific Mentor of Shahab Dental Clinic (Oral Medicine and Special Care Dentistry) Mashhad Iran; ^3^ Department of Radiotherapy Oncology and Cancer Research Center Mashhad University of Medical Sciences Mashhad Iran; ^4^ Department of Oral and Maxillofacial Radiology, Oral and Maxillofacial Diseases Research Center Mashhad University of Medical Sciences Mashhad Iran; ^5^ Department of Oral and Maxillofacial Surgery, Faculty of Dentistry Mashhad University of Medical Sciences Mashhad Iran; ^6^ Student Research Committee, Faculty of Dentistry Mashhad University of Medical Sciences Mashhad Iran; ^7^ Faculty of Dentistry Mashhad University of Medical Sciences Mashhad Iran

**Keywords:** cone‐beam computed tomography (CBCT), denosumab, medication‐related osteonecrosis of the jaw (MRONJ), osteonecrosis, Paget disease

## Abstract

Patients with Paget's disease who receive high‐dose denosumab may develop medication‐related osteonecrosis of the jaw, particularly after dental extractions. Pre‐treatment dental assessment, careful risk communication, accurate staging, and coordinated conservative and surgical management are essential. Platelet‐rich fibrin may be used only as an adjunct, not as an established standard treatment.

## 1 IntroduSction

Paget's disease of bone is a chronic disorder characterized by dysregulated bone remodeling [1], leading to excessive bone resorption followed by disorganized bone formation. This results in abnormal bone architecture, deformities, and an increased risk of fractures and other complications [2]. While the etiology of Paget's disease remains unclear, both genetic and environmental factors are thought to contribute to its development [3]. Paget's disease predominantly affects older adults, with a higher prevalence observed in individuals aged 55 years and older [4].

Medication‐related osteonecrosis of the jaw (MRONJ) is a severe adverse effect commonly linked to antiresorptive medications, including bisphosphonates and denosumab [5]. Recent studies have also implicated antiangiogenic agents in the development of MRONJ, particularly in oncology patients [6]. These medications are commonly prescribed for the treatment of osteoporosis, Paget's disease, and various malignancies [7]. MRONJ is characterized by necrotic bone in the maxillofacial region that persists for more than 8 weeks, which may present as exposed bone or be identified through a fistula, in the absence of radiation therapy to the craniofacial region [5]. While the precise pathogenesis of MRONJ is not fully understood, it is thought to involve impaired bone remodeling, suppressed angiogenesis, and compromised mucosal healing [8].

Reports of MRONJ in patients with Paget's disease are uncommon, and the available evidence is limited mainly to isolated cases and small retrospective observations. A 14‐year retrospective survey identified two patients with Paget's disease among medication‐related osteonecrosis admissions [9]; however, this observation does not establish Paget's disease as an independent risk factor. The altered bone remodeling associated with Paget's disease could theoretically modify susceptibility or clinical presentation, but its contribution remains hypothetical [9, 10]. In patients receiving antiresorptive therapy, medication‐related exposure and local dental triggers should therefore remain central to risk interpretation and prevention [11].

In this case report, we describe medication‐related osteonecrosis of the jaw after multiple dental extractions in a patient with Paget's disease receiving high‐dose denosumab. The report focuses on the diagnostic and management challenges created by this clinical context while distinguishing established risk factors from possible disease‐related modifiers. High‐dose antiresorptive exposure and repeated extractions are considered the most plausible drivers, whereas the independent contribution of Paget's disease cannot be determined.

This case is notable for MRONJ after multiple extractions during high‐dose denosumab therapy, with Paget's disease representing a potentially relevant but unproven modifying context. Management followed a staged conservative and surgical approach; platelet‐rich fibrin was used only as an intraoperative adjunct, and clinical healing was documented at 6 and 8 months.

## 2 Case Presentation

A 55‐year‐old male patient with a known diagnosis of Paget's disease of bone was referred to the Shahab Dental Clinic (Special Care and Oral Medicine Clinic) on February 12, 2024 for evaluation and management. The patient's medical history showed that he had received denosumab 120 mg (XGEVA) every 6 weeks for approximately 1 year, comprising eight injections and a cumulative dose of 960 mg. This dose represents a high‐dose antiresorptive regimen used for oncology‐related skeletal indications rather than standard therapy for Paget's disease; however, the available records did not document the rationale for its selection or indicate whether another clinical indication had been considered [2, 12]. The patient reported a family history of Paget's disease, with his father and paternal uncle previously diagnosed with the condition. The diagnosis was initially established based on complaints of diffuse skeletal discomfort, which correlated with characteristic clinical and radiographic features of Paget's disease.

While receiving denosumab therapy, the patient had several dental extractions. This included the removal of tooth 46 on September 9, 2023, which occurred 1 year after starting XGEVA injections (October 23, 2022). Furthermore, teeth 15, 16, 25, and 26 were extracted in the last 6 months. One month after the last extraction, he presented with non‐healing sockets. MRONJ risk had not been considered at the time of extraction. As a result, the patient underwent multiple extractions without any consideration or warning regarding this potential complication.

### 2.1 Differential Diagnosis

Based on clinical presentation and medication and dental history, a preliminary diagnosis of MRONJ was made. A cone‐beam computed tomography (CBCT) scan was requested and reviewed for a more precise radiographic examination to facilitate diagnosis (Figure 1).

**FIGURE 1:**
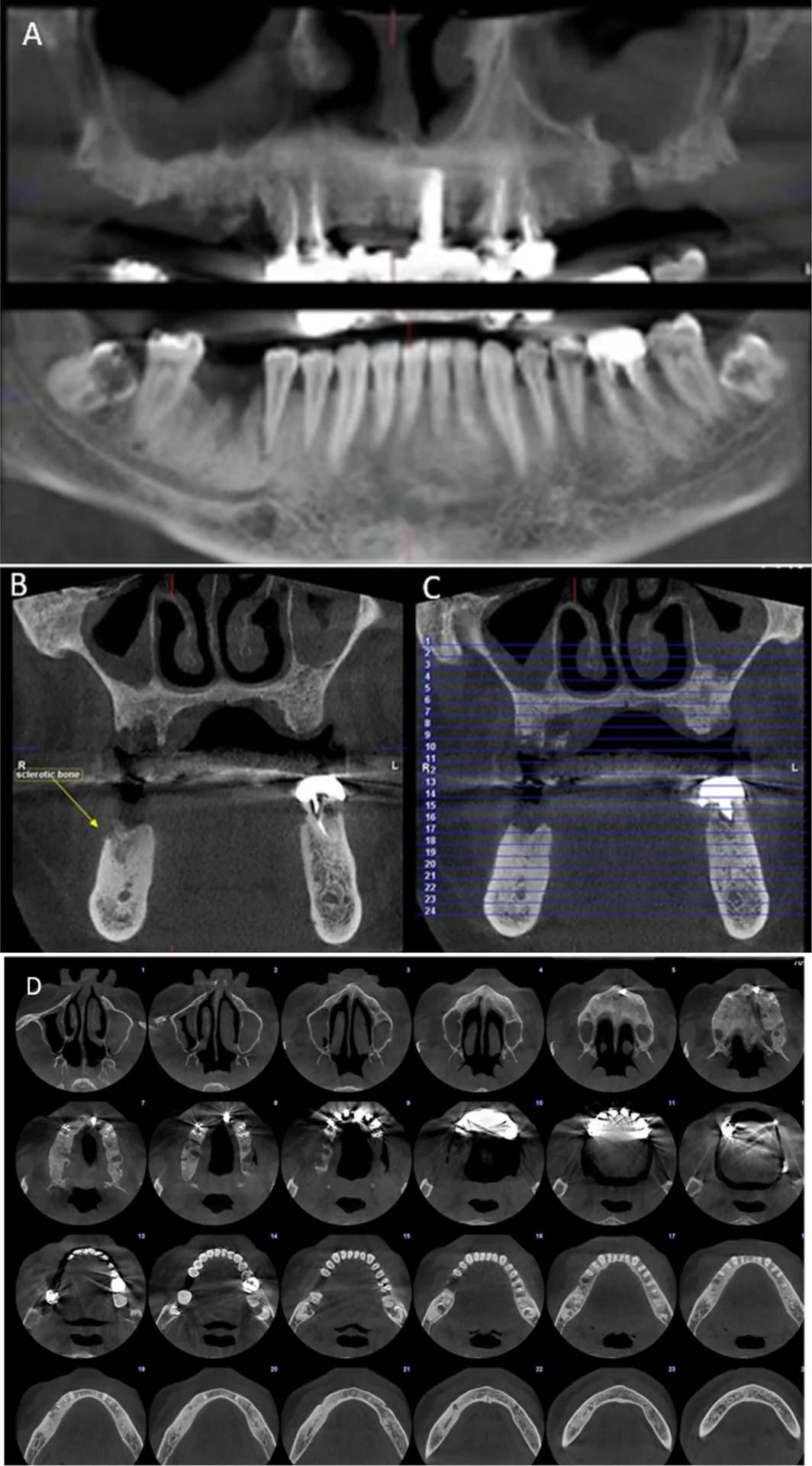
Preoperative cone‐beam computed tomography findings. (A) Reformatted panoramic reconstruction showing generalized increased jawbone density and altered trabecular architecture. (B) Coronal/cross‐sectional view through the right posterior mandible showing focal alveolar sclerosis at an extraction site (arrow). (C) Cross‐sectional/coronal reconstruction used to assess the posterior jaw and extraction‐site sclerosis. (D) Serial axial slices demonstrating generalized increased density with focal sclerotic changes in the posterior jaws. The localized sclerotic changes at extraction sites were considered supportive of medication‐related osteonecrosis of the jaw in the clinical context; no periosteal reaction is evident in the displayed regions.

The reformatted panoramic reconstruction (Figure 1A) shows generalized increased jawbone density and fine, condensed trabeculae with a granular appearance, compatible with the patient's Paget's disease. Coronal/cross‐sectional CBCT images (Figure 1B,C) demonstrate focal increased alveolar density/sclerosis at the right mandibular extraction site and other posterior extraction regions. Serial axial slices (Figure 1D) further demonstrate the generalized density alteration and focal sclerotic changes. No periosteal reaction is evident in the displayed regions. In combination with the clinical findings and antiresorptive history, the localized sclerosis at extraction sites supports the diagnosis of medication‐related osteonecrosis of the jaw.

A risk assessment for dental treatment was conducted, leading to a consultation with an oncologist for further evaluation and management. Based on the findings, a two‐phase treatment plan was proposed.

Diagnosis & staging: Findings fulfilled AAOMS (2022) criteria for MRONJ (exposed bone ≥ 8 weeks, antiresorptive exposure, no head/neck radiotherapy), and the lesion was categorized as Stage 2. Biopsy was not performed to avoid lesion exacerbation; clinico‐radiographic features were sufficient for diagnosis and to exclude mimics. Typical MRONJ histology includes necrotic lamellar bone with empty lacunae and surface bacterial colonization; these features were not sampled in this case.

### 2.2 Outcome and Follow‐Up

The first phase, initiated on the same date, included amoxicillin 500 mg three times daily for 14 days, pentoxifylline 400 mg twice daily for 8 weeks, vitamin E 400 IU daily for 8 weeks, and chlorhexidine 0.12% mouthwash twice daily for 4 weeks, prescribed after oncologist approval. Additionally, calcium supplementation was recommended for the patient by the oncologist.

For the second phase, the patient was referred to an oral and maxillofacial surgeon. This phase involved a follow‐up evaluation after 2 weeks and potential adjustment of the treatment regimen based on the patient's response. It was noted that XGEVA injections had been discontinued as per the physician's recommendation on November 23, 2023. In the continuation of the second phase, the patient was referred to the surgery department of Mashhad Dental Faculty. During the clinical examination, exposed necrotic bone was observed in the regions of teeth 16, 26, and 46, as shown in Figure 2. There were no signs of active infection present. Based on these findings, a diagnosis of MRONJ Stage 2 was established according to the staging criteria proposed by the American Association of Oral and Maxillofacial Surgeons (AAOMS).

**FIGURE 2:**
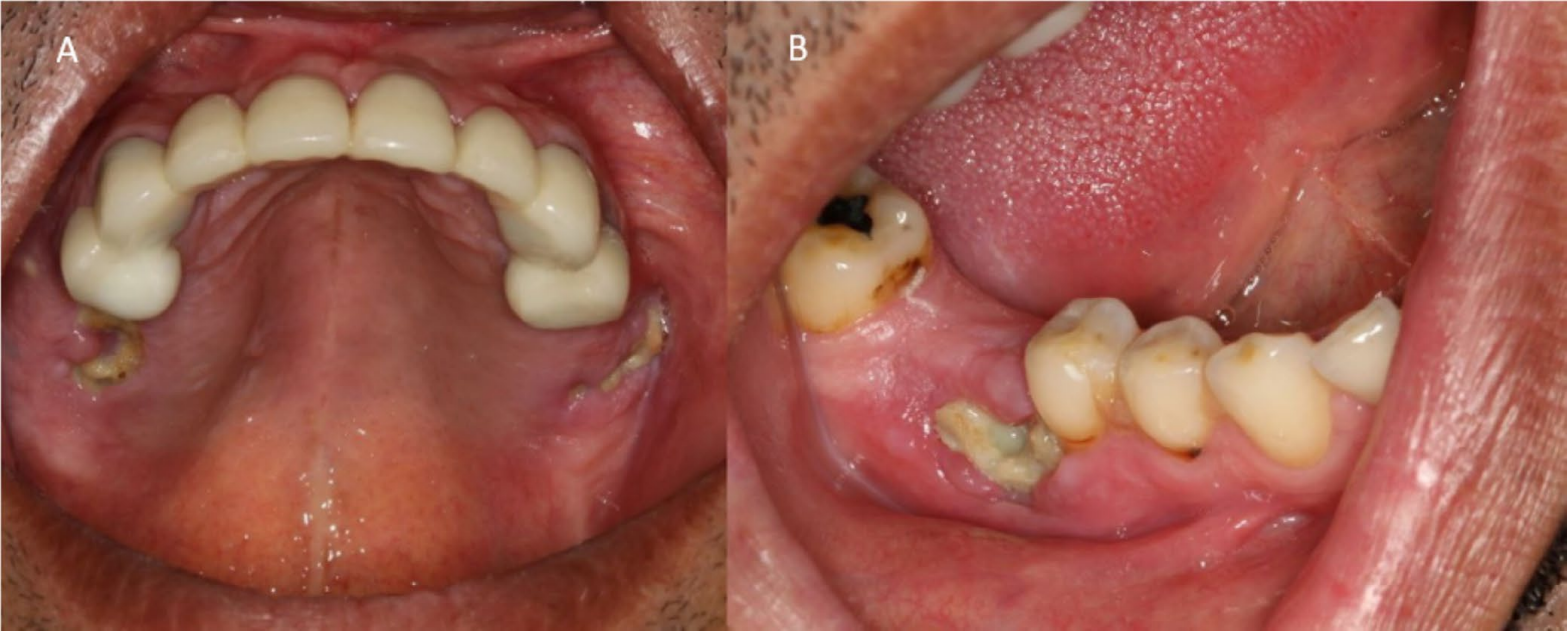
Preoperative intraoral images. (A) Maxillary extraction sites showing exposed necrotic bone. (B) Right mandibular extraction site showing exposed necrotic bone. Surrounding mucosal changes are present without purulent discharge.

The surgical phase consisted of debridement of the necrotic bone and advancement of a buccal flap to obtain soft‐tissue coverage. Platelet‐rich fibrin was prepared by centrifuging 10 mL of the patient's blood without anticoagulants, producing an autologous fibrin clot containing platelets and leukocytes. It was selected as an adjunct because the fibrin matrix was expected to support clot stability and soft‐tissue closure at the debrided sites. Platelet‐rich fibrin was not used as a substitute for debridement, antimicrobial measures, or tension‐free flap closure, and its independent contribution to healing cannot be determined from this case [13].

At 6 months (September 19, 2024), pain had resolved, the mucosa had re‐epithelialized, and no suppuration or fistula was evident. At 8 months (November 19, 2024), clinical stability persisted; follow‐up imaging, not shown in the present figure set, demonstrated interval improvement. The patient nevertheless reported intermittent lower‐extremity bone pain. The oral and maxillofacial surgeon therefore recommended reassessment with the treating physician regarding resumption of denosumab (XGEVA, 120 mg) in view of the ongoing systemic skeletal symptoms and the favorable local healing. The records available for this report document the recommendation but do not confirm whether denosumab was subsequently restarted or whether dose modification or an alternative antiresorptive strategy was formally considered.

At the 8‐month visit, further clinical improvement was present, with no recurrent infection or progression of the necrotic lesions. A small papule‐like mucosal lesion was noted; however, there was no associated fistula or purulent discharge (Figure 3).

**FIGURE 3:**
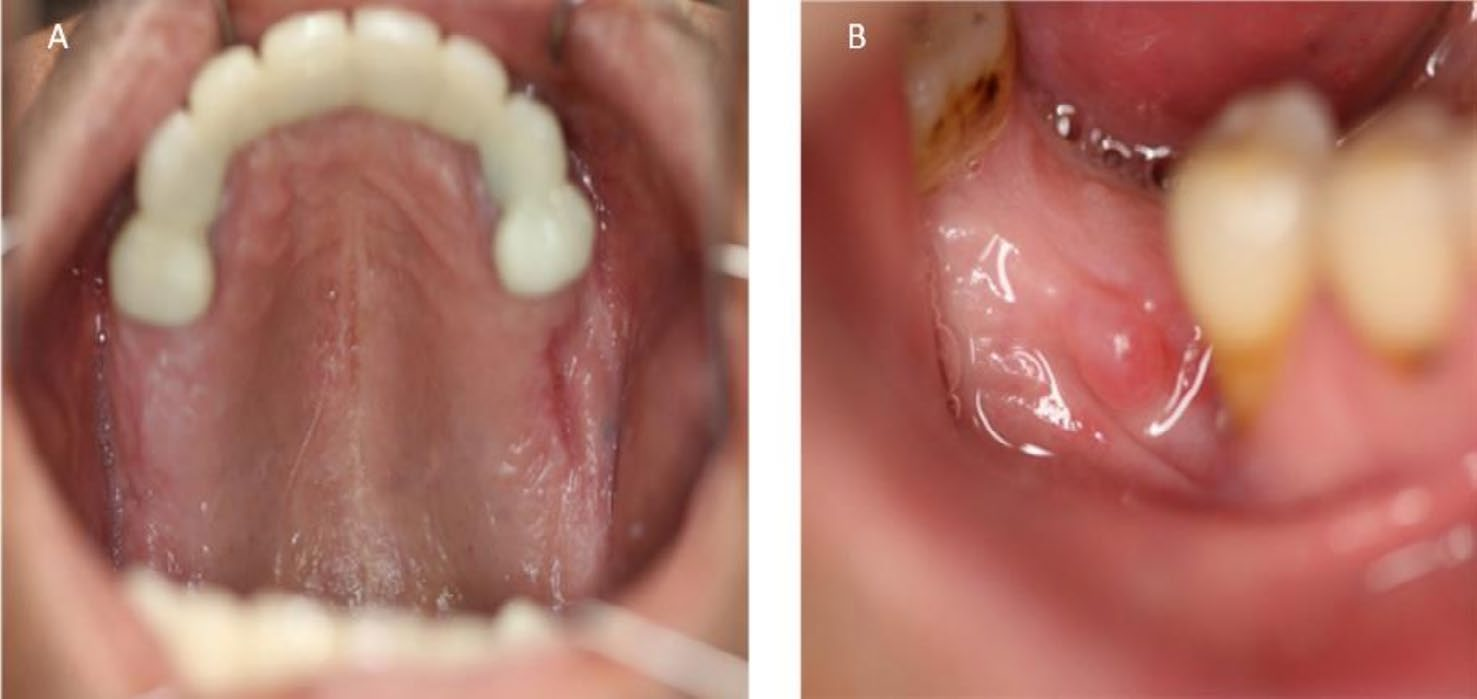
Follow‐up intraoral photographs obtained during the 6‐ and 8‐month follow‐up period. (A) Maxillary mucosal healing. (B) Mandibular mucosal healing. Both panels show mucosal coverage without purulent discharge; the small papule‐like area in panel B was not associated with a fistula.

## 3 Discussion

Since the first description in 2003, multiple drug classes have been associated with MRONJ. A significant aspect of the epidemiology of MRONJ is the different medical conditions that may necessitate the prescription of these medications. It is crucial for dentists to be aware of when and under which medical conditions they might anticipate the occurrence of MRONJ in their clinical practice.

This case report describes a rare instance of a patient diagnosed with Paget's disease of bone who later developed MRONJ. The combination of these two conditions required a multidisciplinary approach, involving consultations with specialists in oral medicine, oncology, and oral and maxillofacial surgery for thorough evaluation and management.

Magremanne et al. reported a similar case of MRONJ in a patient with Paget's disease receiving bisphosphonate therapy. Their study highlighted how recurrent episodes of osteonecrosis coincided with ongoing bisphosphonate administration, emphasizing the importance of preventive measures such as pre‐treatment dental clearance and regular monitoring during therapy. The report demonstrated the need for vigilance in managing patients with Paget's disease undergoing antiresorptive treatment, especially considering the higher risks associated with jaw involvement in this population [14]. The management of MRONJ in our case and in Magremanne et al.'s report involved a combination of conservative and surgical interventions. While Magremanne et al. focused on debridement and local wound care during episodes of MRONJ, our case utilized advanced imaging modalities like CBCT and adjunctive therapies such as platelet‐rich fibrin (PRF) to enhance healing. These distinctions highlight the evolving approaches to MRONJ management, with newer techniques showing promise in improving patient outcomes. Preventive strategies remain crucial: pre‐treatment dental clearance, regular oral assessment during therapy, and patient education about MRONJ risk, particularly when invasive procedures are planned.

MRONJ is primarily associated with antiresorptive exposure, including bisphosphonates and denosumab [5, 15]. Denosumab inhibits receptor activator of nuclear factor kappa‐B ligand and suppresses osteoclast‐mediated bone resorption [16]. In this patient, the 120 mg regimen and cumulative exposure of 960 mg should be regarded as major medication‐related risk factors, particularly in the presence of multiple dental extractions. The dose is characteristic of the high‐dose XGEVA treatment context used for oncology‐related skeletal indications, whereas guidelines for active Paget's disease recommend bisphosphonate therapy rather than high‐dose denosumab [2, 12]. The every‐six‐week interval also differs from the usual monthly XGEVA schedule described for metastatic bone disease [12]. Because the prescribing records were unavailable, the rationale for this regimen cannot be reconstructed. Plausible explanations include an undocumented oncologic or bone‐tumor indication, concern about malignant transformation of Paget's disease, or off‐label/non‐standard prescribing. The involvement of an oncologist and calcium supplementation indicate specialist oversight but do not establish any of these possibilities. Importantly, the material available for this report did not document a primary malignancy, metastatic disease, multiple myeloma, or Paget‐associated sarcoma; these possibilities are therefore discussed only as explanations for the unusual prescription and not as diagnoses. The most defensible interpretation remains that high‐dose denosumab exposure combined with repeated extractions was the dominant documented MRONJ risk context, while Paget‐related alterations in bone remodeling may have acted only as a possible, unproven modifier [1, 2, 12, 15].

In diagnosing MRONJ, we followed the guidelines from AAOMS, using clinical and radiographic evaluations to establish MRONJ at Stage 2 [5, 12]. However, diagnosis in cases complicated by concurrent Paget's disease can be challenging due to overlapping symptoms and altered bone morphology [10]. Advanced imaging techniques like CBCT can provide critical insights into bone quality and areas of necrosis in such complex cases, assisting in staging and guiding surgical planning [17]. Studies show that the AAOMS staging system, while effective, can sometimes require modification in patients with pre‐existing bone disorders like Paget's, where bone quality is compromised and osteonecrosis may present atypically [18].

The management of MRONJ necessitates a multidisciplinary approach [8], involving both conservative and surgical interventions. Conservative measures typically include antimicrobial therapy and oral rinses [8, 19]. In our case, the initial regimen consisted of amoxicillin, pentoxifylline, vitamin E, and chlorhexidine as detailed in outcome and follow‐up [20, 21]. The role of an antiresorptive drug holiday is controversial rather than routine: the 2022 AAOMS position paper states that evidence supporting or refuting drug holidays remains inconclusive and recommends individualized consideration of disease‐related risk, dose, treatment duration, and surgical factors [12]. Denosumab requires particular caution because its antiresorptive effect is reversible and rebound bone resorption with increased vertebral fracture risk has been described after discontinuation, particularly in osteoporosis populations; interruption and re‐initiation should therefore be coordinated with the prescribing clinician [12]. In this patient, lower‐extremity bone pain appeared after denosumab had been discontinued. This symptom could have reflected activity of the underlying skeletal disease or a post‐denosumab rebound state, but pain alone is nonspecific and no biochemical or imaging evidence was available to establish either explanation. The later recommendation to consider resuming XGEVA after local healing was therefore interpreted as an individualized systemic risk–benefit decision, not as evidence that a drug holiday is generally beneficial or that denosumab should routinely be restarted after MRONJ. No documentation showed that dose reduction or substitution with another antiresorptive agent was formally considered; moreover, alternative antiresorptives also carry MRONJ risk, so any such modification would require coordination with the treating physician [12, 18].

Following conservative therapy, the patient underwent surgical debridement, with platelet‐rich fibrin applied only as an adjunct. The intended benefit was to provide an autologous fibrin scaffold that might support clot stability and soft‐tissue healing after removal of the necrotic bone. Small clinical studies have reported favorable outcomes when platelet‐rich fibrin is combined with surgical treatment [22, 23]; however, these findings do not establish it as a standard or independently effective treatment for MRONJ. The AAOMS' stage‐based recommendations emphasize antimicrobial care and appropriate surgical management rather than platelet‐rich fibrin as a required treatment component [12]. The favorable outcome in this patient should therefore be interpreted as the result of the overall staged strategy, including antimicrobial care, debridement, flap closure, and follow‐up, rather than platelet‐rich fibrin alone.

Preventive strategies are paramount for reducing MRONJ incidence in high‐risk patients on antiresorptive therapies, especially those with Paget's disease [18, 24].

The most effective strategy is to achieve dental clearance before starting medications that may increase the risk of MRONJ. It includes conducting extractions and invasive procedures before initiation of medications, maintaining regular oral hygiene, and addressing any oral infections promptly can help prevent MRONJ. Additionally, avoiding elective invasive dental procedures during medication administration and performing antibiotic prophylaxis and antimicrobial mouth rinses can help to deliver safe surgical treatment to patients. Prophylactic antibiotics, though debated, are considered beneficial in certain high‐risk dental procedures for MRONJ‐prone patients, with evidence suggesting reduced MRONJ occurrence when used preemptively [2]. In the context of Paget's disease, interdisciplinary monitoring is critical [1, 18]. A collaborative approach involving dental surgeons, maxillofacial specialists, and primary care providers ensures a cohesive management plan [18].

Our stage‐based approach aligns with AAOMS 2022 guidance, emphasizing conservative measures for Stage 2 with selective debridement and close follow‐up [12, 18, 19, 20, 21].

### 3.1 Practice Flow

Complete dental assessment before antiresorptive therapy → avoid elective extractions during antiresorptive therapy when clinically feasible → if exposed or probeable bone persists for at least 8 weeks, stage according to the AAOMS criteria → for Stage 2 disease, provide an antimicrobial mouth rinse, systemic antibiotics when clinically indicated, and surgical evaluation → perform debridement or resection as appropriate → coordinate follow‐up with the prescribing physician. In this case, platelet‐rich fibrin was used as an optional surgical adjunct and is not included as a standard step in the proposed management pathway.

MRONJ in this patient most plausibly reflected high‐dose denosumab exposure combined with repeated dental extractions. Paget's disease may have modified the local bone response, but its independent contribution remains uncertain. Pre‐treatment dental assessment, careful staging, and coordinated conservative and surgical care are essential for reducing risk and supporting healing.

## Author Contributions

**Toktam Zamani:** conceptualization, investigation, project administration, resources, supervision, writing – original draft. **Pegah Mosannen Mozaffari:** conceptualization, data curation, project administration, resources, supervision. **Kazem Anvari:** project administration, resources, supervision, writing – review and editing. **Najmeh Anbiaee:** project administration, resources, supervision, writing – review and editing. **Khashayar Famili:** project administration, resources, supervision, writing – review and editing. **Ali Mirzaei:** investigation, visualization, writing – original draft. **Fatemeh Fallah Toosi:** project administration, writing – review and editing. **Abolfazl Shafiee:** data curation, visualization, writing – review and editing.

## Funding

The authors have nothing to report.

## Consent

Written informed consent was obtained from the patient for publication of this case report and all accompanying anonymized clinical information and images.

## Data Availability

The de‐identified clinical information underlying this case report is available from the corresponding author upon reasonable request; public sharing is restricted to protect patient privacy.
